# Small activating RNA induces myogenic differentiation of rat adipose-derived stem cells by upregulating MyoD

**DOI:** 10.1590/S1677-5538.IBJU.2014.0400

**Published:** 2015

**Authors:** Chenghe Wang, Zhong Chen, Jia Wu, Yan Zhang, Jia Hu, Qiangqiang Ge, Tao Wang, Weimin Yang, Hua Xu, Jihong Liu, Zhangqun Ye

**Affiliations:** 1 Department of Urology, Tongji Hospital, Tongji Medical College, Huazhong University of Science and Technology, Hubei, China

**Keywords:** RNA, MyoD Protein, Urinary Bladder Neoplasms, Urinary Incontinence, Stress, Desmin

## Abstract

**Purpose:**

RNA activation (RNAa) is a mechanism of gene activation triggered by promoter-targeted small double stranded RNAs (dsRNAs), also known as small activating RNAs (saRNAs). Myogenic regulatory factor MyoD is regarded as the master activator of myogenic differentiation cascade by binding to enhancer of muscle specific genes. Stress urinary incontinence (SUI) is a condition primarily resulted from urethral sphincter deficiency. It is thus expected that by promoting differentiation of adipose-derived stem cells (ADSCs) into myoblasts by activating MyoD gene through RNAa may offer benefits to SUI.

**Materials and Methods:**

Rats ADSCs were isolated, proliferated in vitro, and identified by flow cytometry. Purified ADSCs were then transfected with a MyoD saRNA or control transfected. Real-time polymerase chain reaction (RT-PCR) and western blotting were used to detect MyoD mRNA and protein expression, respectively. Immunocytochemical staining was applied to determine the expression of desmin protein in transfected cells. Cell viability was measured by using CellTiter 96® AQ_ueous_ One Solution Cell Proliferation Assay kit.

**Results:**

Transfection of a MyoD saRNA (dsMyoD) into ADSCs significantly induced the expression of MyoD at both the mRNA and protein levels, and inhibited cell proliferation. Desmin protein expression was detected in dsMyoD treated ADSCs 2 weeks later.

**Conclusion:**

Our findings show that RNAa mediated overexpression of MyoD can promote transdifferentiation of ADSCs into myoblasts and may help treat stress urinary incontinence (SUI)–a condition primarily resulted from urethral sphincter deficiency.

## INTRODUCTION

Small double stranded RNAs (dsRNAs) are known to be able to induce sequence-specific genes expression by targeting gene promoter regions, a phenomenon known as RNA activation (RNAa) and thus named as small activating RNA (saRNAs) (1). Li and colleagues have demonstrated that saR-NAs could activate genes E-cadherin, p21^WAF1/CIP1^ and VEGF expression in human cell lines (1). Our previous study also confirmed that saRNA could elicit antitumor activity by triggering the expression of p21^WAF1/CIP1^ in human bladder cancer cell lines (2). Moreover, other groups have since reported similar outcomes in human cells (3) and in other mammalian species as well (4,5).

It is well known that RNA interference (RNAi) is being actively pursued as therapeutics for many human diseases owing to its down-regulation of a particular gene expression. However, some diseases, especially related to tissue degeneration or damage, are often caused by decreased expression of certain gene products involved in crucial physiological function (6). These defects are difficult to be cured by RNAi and may be restored by RNAa.

Some urologic diseases involving kidney, bladder and urethra are caused by specific tissue damage (7–9). Especially, stress urinary incontinence (SUI) is a common disorder mainly resulted from the support tissue deficiency, such as smooth muscle damage (10). Recent advances in tissue engineering indicated that transurethral injection of adipose-derived stem cells (ADSCs) could improve anatomic abnormality and symptoms of SUI (11–13). Moreover, a small fraction of ADSCs might differentiate into smooth muscle, but the majority appeared to remain undifferentiated (12). To enhance ADSCs myogenic differentiation may further recover urethral function and ameliorate SUI.

MyoD, a member of the myogenic regulatory factors (MRFs) family, is a basic helix-loop-helix DNA binding transcription factor and has often been referred as the master regulator of myogenesis (14,15). The family has other three members: Myf-5, Myogenin, and MRF-4. MRFs have a critical property that they can convert many cell types into myogenesis and MyoD shares an overlapping function with myf-5 for generating muscle cell identity and activating myogenin (15). In addition, Kocaefe et al. have presented that a terminally differentiated mature adipocyte possessed the proliferative capacity which could commit to a myogenic program by MyoD manipulated (16). Hereby, we picked MyoD as the specific target for saRNAs.

In the present study, 5 specific dsRNA candidates targeting MyoD gene promoter were designed and transfected into rat ADSCs. We investigated the MyoD gene expression, ADSCs’ myogenic differentiation and cell proliferation. Our results show that a candidate dsMyoD-373 potently upregulated MyoD expression and induced rat ADSCs differentiated into myoblasts. This method would be useful in enhancing the therapeutic efficacy of ADSCs for SUI.

## MATERIALS AND METHODS

### dsRNA design and synthesis

The candidates of dsRNAs were rationally designed after 1 kilobase of the rat MyoD promoter sequences was scanned for saRNA target sites, based on the rule as previously described (1,17). In addition, a dsRNA lacking significant homology to all known rat sequences (dsControl) was used as a non-specific control. The specific sequences of dsControl were provided and all the synthetic dsRNAs were manufactured by Ribo bio (Guangzhou, China).

### Rat ADSCs isolation and culture

Isolation, culture and passage of rat ADSCs were performed as previously described (13). Briefly, rat adipose tissue was collected from inguinal fat pad without muscle contamination. Adipose tissue was washed extensively with phosphate buffered saline (PBS) (Gibco, California, USA) and minced into small pieces, and then digested with 0.1% collagenase type I solution (Sigma, California, USA) at 37°C for 30 minutes. After filtered through 200μm stainless steel mesh, the cells were centrifuged and then resuspended in high-glucose Dulbecco's modified Eagle's medium (HyClone Inc., Massachusetts, USA) containing 10% fetal bovine serum (Gibco, California, USA). Next, the cells were incubated with 5% CO_2_ at 37°C. After 24 hours, unattached cells and debris were removed, and fresh medium was added to the adherent cells. Cells were passaged when they reached 80% confluence. The animal experimental procedures were approved by the Institutional Animal Care and Use Committee (IACUC) of the Tongji Medical College of Huazhong University of Science and Technology.

### Rat ADSCs identification

Cells of the fourth generation in logarithm growth phase were digested and cell density was adjusted to approximately 1×10^6^/mL. The cell suspension was analyzed by flow cytometry to detect ADSC-specific antigens. Antibodies used in the experiment included anti-CD31, CD49, CD90, CD106 (BD biosciences, New Jersey, USA), CD34 (Santa Cruz, Texas, USA), CD45 (AbD Serotec, North Carolina, USA), CD73 and CD105 (Bioss, Beijing, China). The data was analyzed using CellQuest software (BD Biosciences, New Jersey, USA).

### dsRNA transfection

Immediately before transfection, ADSCs were plated in 6-well plates with growth medium without antibiotics (approximate 2.5×10^5^ cells for each well). Reverse transfection of dsRNAs was carried out using Lipofectamine^TM^ RNAiMax (Invitrogen, California, USA) according to the manufacturer's instructions. The final concentration of dsRNA of each well was 50nM. Additionally, dsRNA was replaced by MEM in mock transfection. The medium containing transfection reagent was replaced by normal medium 8 hours later and then medium was changed daily. The cells were harvested at certain time following transfection and subjected to scheduled experiments.

### RNA extraction and real-time PCR analysis

Total cellular RNA from rat ADSCs was extracted by using Trizol reagent (Invitrogen, California, USA). Then, first-strand complementary DNA (cDNA) was synthesized from 500ng of RNA according to the protocol provided by Takara reverse transcription kit (Takara, Dalian, China). Real-time quantitative PCR was performed on the Mx3000P system (Stratagene, California, USA) using SYBR Premix Ex Taq^TM^ II (Takara, Dalian, China) according to the manufacturer's instructions. The primers (Invitrogen, California, USA) for cDNAs were used as follows: MyoD 5’-GGAGACATCCTCAAGCGATGC-3’ (F) and 5’-AGCACCTGGTAAATCGGATTG-3’ (R); GAPDH 5’-CCACCAACTGCTTAGCACC-3’ (F) and 5’-GCCAAATTCGTTGTCATACC-3’ (R). Amplification was performed under the following cycling conditions: an initial denaturation at 95°C for 30 seconds, then 40 cycles of denaturation at 95°C for 5 seconds, annealing at 55°C for 30 seconds, and extending at 72°C for 30 seconds. Amplification of GAPDH was used to normalize target gene's expression level. All samples were measured in triplicate.

### Protein isolation and Western blot analysis

Total proteins were extracted from rat ADSCs using NP40 lysis buffer supplemented with protease inhibitor phenylmethanesulfonyl fluoride (PMSF) (Missouri, USA). The protein concentration was determined using a BCA protein assay (Beyotime, Shanghai, China). For each group, 50μg samples were taken, and subjected to 10% sodium dodecyl sulfate polyacrylamide gel (SDS-PAGE) electrophoresis and then transferred to a PVDF membrane. Membranes were blocked in 5% nonfat dried milk. After several washes with washing buffer, the membranes were incubated with the primary antibodies (mouse Anti-MyoD1 antibody) overnight at room temperature 4° C. The primary antibodies (monoclonal antibodies) were as follows: (i) MyoD (1/400) (Abcam, Massachusetts, China) and (ii) GAPDH (1/1000) (Boster, Wuhan, China). The washed membranes were incubated for 2 hours at room temperature with 5000-fold diluted horseradish peroxidase (HRP)-conjugated goat anti-mouse IgG antibody. After several washes, immunodetected proteins were visualized by using enhanced chemiluminescence (ECL) (Thermo, Massachusetts, USA) assay kit. Optical density from Western blotting assay was quantified with BandScan 5.0 software.

### Immunocytochemistry

Rat ADSCs transfected with dsRNAs for 7, 14, 21, and 28 days were subjected to immunocytochemical staining, respectively. The cells were plated at a density of 1×10^4^ cells/cm^2^ in coverslips which were placed in culture plates and cultured overnight. The cells were fixed with 4% paraformaldehyde for 15 minutes and permeated for 10 minutes with 0.5% Triton X-100 diluted with PBS. The endogenous peroxidase activity was quenched with 3% hydrogen peroxidase in methanol for 15 minutes. Then an antibody directed against rat desmin (Bioworld, Minnesota, USA) at a dilution of 1:150 was added overnight at 4°C followed by the polymer helper and polyperoxidase-anti-mouse/rabbit IgG (Boster, Wuhan, China) incubated for 20 minutes. All incubations were conducted at room temperature and slides were rinsed three times for 3 minutes in PBS between steps. Diaminobenzidine (DAB) acted as the chromogen. After counterstained with Harris hematoxylin, dehydrated with graded alcohols, bathed in fresh xylene and covered with gummi, cells were visualized under a light and positivity for desmin in the cytosol were stained brown in different hues.

### Cell growth/viability assay

Proliferation of cells was detected by the CellTiter 96® AQ_ueous_ One Solution Cell Proliferation Assay kit (Promega, Madison, USA). ADSCs were seeded in a 96-well plate with cell density of approximate 5000 cells per well. The dsRNAs were transfected into cells at a final concentration of 50nM following reverse transfection. The plates then were incubated for 5 days and cell growth was measured at 5 points every 24 hours from day 1 to day 5 after transfection. At each time point, culture medium of the 96-well plates was replaced by 10μL CellTiter 96® AQ_ueous_ One Solution premixed with 100μL fresh medium. Followed by 2 hours incubation at 37°C, absorbance was determined by an absorbance reader (Thermo, Massachusetts, USA) at 490nm. The reduction in viability of each group was expressed as a percentage of the mock group, which was considered to be 100% viable.

### Statistical analysis

Data were statistically analyzed using SPSS version 13.0 software (SPSS Inc., Chicago, IL, USA). All data are presented as mean±standard deviation (SD) for three independent experiments. Differences among groups were analyzed by student's t tests. P values less than 0.05 were considered statistically significant.

## RESULTS

### ADSCs morphology and surface antigens

**Figure 1:**
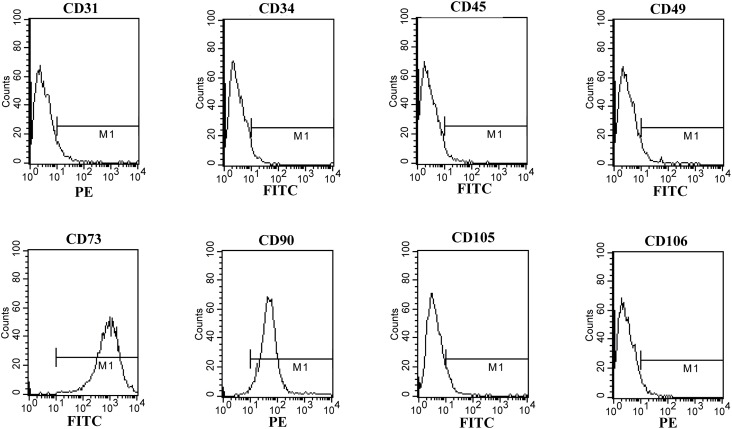
Flow cytometry analysis of rat ADSCs. Cells were analyzed by flow cytometer after staining with PE-or FIFC-conjugated antibodies against indicated cell surface proteins.

After 2 days, the non-adherent cells were rinsed off by changing the culture medium, and a few cells adhered to culture flask. Initially, adherent cells shape presented to be irregular, short spindle-like or stellate. Afterwards, cells gradually developed into uniformly spindle-like throughout the bottom and began to proliferate rapidly. Moreover, the cells spread evenly and showed less alteration in appearance after passage 2. Flow cytometry analysis revealed the cells had a high level of CD73 expression (99.88%), CD90 (98.74%) and CD105 (5.89%), but no significant expression of CD31 (6.64%), CD34 (4.12%), CD45 (3.15%), CD49 (5.07%) or CD106 (3.93%) (Figure-1). These results were similar to a previous study (18).

### MyoD gene activation in rat ADSCs by promoter targeted saRNA

**Figure 2:**
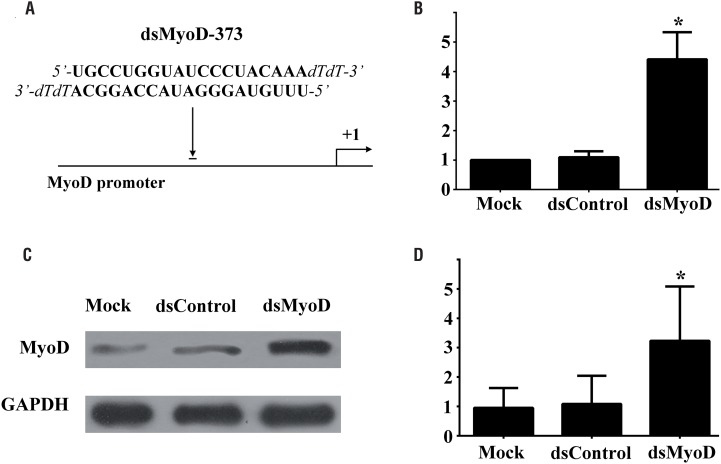
MyoD gene expression induced by dsMyoD in ADSCs. (A) A schematic representation of the MyoD promoter, its transcription start site and the location of the saRNA target. (B) Induction of MyoD mRNA expression. (C) Induction of MyoD protein expression. GAPDH served as a loading control. (D) Densitometric analysis quantification of MyoD protein expression. Cells were transfected with 50nM dsRNA for 72 hours. *P<0.05 versus dsControl transfected group and mock group.

Rat ADSCs were transfected with candidate dsRNAs or dsControl at a concentration of 50nM, or mock transfected for 72 hours, and the expression of MyoD mRNA and protein was evaluated by Real-time PCR and Western blotting analysis, respectively. The outcomes indicated that a 21-nucleotide (nt) dsRNA candidate targeting the MyoD gene promoter at position-373 relative to the transcription start site (dsMyoD-373) had the ability to activate MyoD expression (Figure-2A). The expression of MyoD was significantly induced at mRNA level by 4.42-fold (P<0.05) and 4.03-fold (P<0.05) compared to mock and control transfection, respectively (Figure-2B). To further confirm the induction at mRNA level, we performed western blotting analysis, dsMyoD transfection led to a 3.39-fold (P<0.05) and 2.98-fold (P<0.05) increase in protein expression compared with mock and group control, respectively (Figure 2C and 2D).

### Desmin protein expression of rat ADSCs following transfection

**Figure 3:**
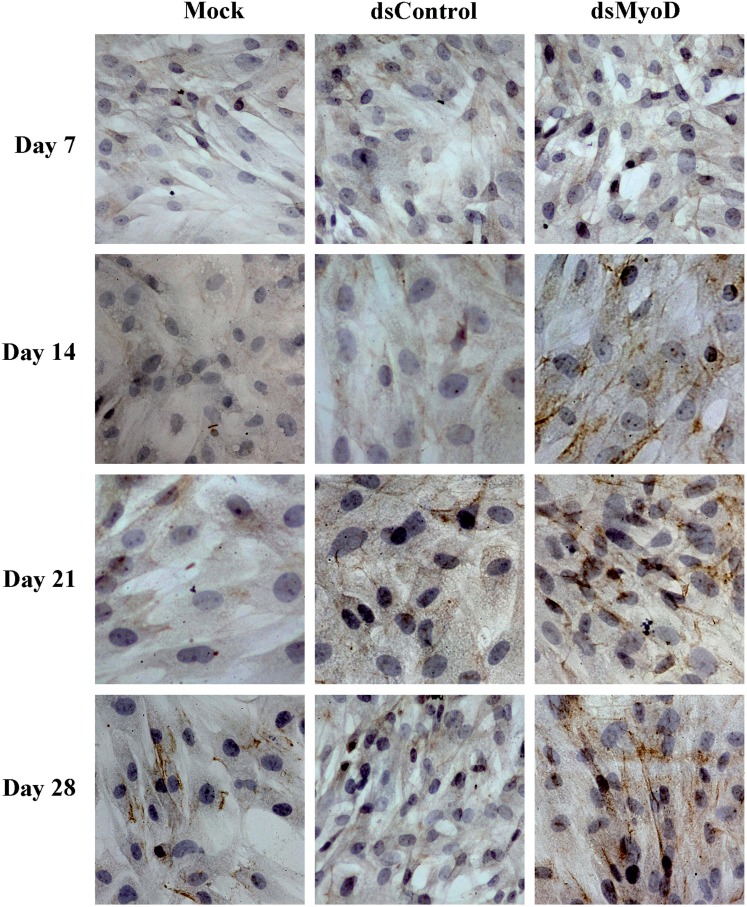
Immunocytochemical analysis of desmin protein expression in ADSCs following transfection of dsMyoD at different time points. ADSCs were mock transfected or transfected with the indicated dsRNAs at 50nM. Expressed cytoplasmic desmin protein was stained brown in cell images. The magnification is×400.

As the muscle-specific member of intermediate filaments, cytoskeletal protein desmin is expressed in all muscle tissues. And desmin is known as one of the most important myogenic markers (19). In this research, before transfection of dsMyoD, desmin protein was not observed in cytoplasm of ADSCs by immunocytochemistry staining (data not shown). After 7 days of transfection, desmin protein expression was not detected in any groups. But at 14 days, it was detected in dsMyoD group. After 21 days of transfection, the cytoplasm stained positive significantly for desmin protein, and this representation was even more obvious at 28 days. However, desmin protein was never observed in Mock or dsControl group (Figure-3).

### Inhibition of rat ADSCs proliferation by dsMyoD transfection

**Figure 4:**
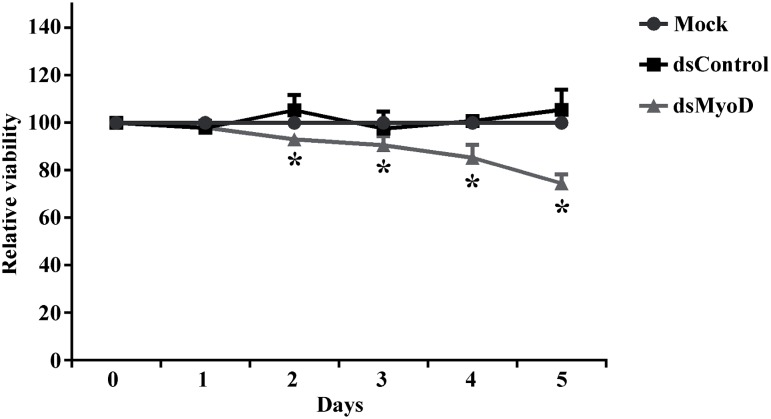
MyoD saRNA inhibits proliferation of rat ADSCs. Cells were transfected with 50nM of indicated dsRNA or mock transfected. *P<0.05 versus mock transfection.

To quantitatively measure cell proliferation rate, we performed cell proliferation assays with ADSCs following mock transfection or transfection with indicated dsRNA. As illustrated in Figure-4, cells transfected with dsMyoD exhibited progressive retarded growth compared to mock transfection or dsControl transfection. Meanwhile, cells transfected with dsControl possessed similar growth as mock transfected cells. By day 5, ds-MyoD transfected cells exhibited a 25.4% (P<0.05) reduction in viable cells compared to mock.

## DISCUSSION

In this study we demonstrated dsMyoD-373 had the capacity to enhance MyoD gene expression and induce ADSCs myogenic differentiation in vitro. Contrary to RNAi, RNAa is a fundamental and promising discovery of dsRNAs which triggers gene up-regulation by targeting promoter sequences originally identified in human cells (2,20). Although its underlying molecular mechanism remains elusive, it is reasonable to believe that small RNA-mediated gene regulation may have evolved to the capacity to regulate gene expression both negatively and positively (21). In this regard, some muscle deficiency related diseases resulted from insufficient expression of functional gene can be cured.

The saRNAs have a target size of 19 nt with dTdT overhangs which is similar to classic small interfere RNAs (siRNAs) (1). However, mismatches in regions outside the seed sequences are tolerable and preserve partial RNAa activities (22). The lasting period of RNAa was much longer than RNAi (21,23). The effect of RNAi can last for only 5-7 days, while RNAa can maintain effective for nearly two weeks (1,23). In the present study, the cytoskeletal protein desmin exhibited persistent expression after single transient transfection of dsMyoD-373.

Previous studies had shown that ADSCs can be induced to differentiate into myoblasts by 5-azacytidine and desmin protein were detected positive 21 days after induction (11,24). However, in present study, the desmin protein was detected at 2 weeks after transfection of dsMyoD, and the expression gradually increased at 3 and 4 weeks. Moreover, ADSCs induced by 5-azacytidine recovered urethral function faster and more significantly compared to no induction (11). Thus, activation of myogenic regulatory factor MyoD promoted ADSCs differentiation earlier and might help to improve urethral function at a greater extent.

A variety of studies revealed that MyoD contributed much to restore urethral sphincter by periurethral injection of muscle precursor cells or stem cells (25,26). Moreover, Liu and colleagues reported that human urine-derived stem cells could express myogenic markers (MyoD, myf-5 and desmin) at 4 weeks after subcutaneously implanted into nude mice, and this approach has a crucial potential for correcting sphincter muscles impairment of SUI (27). Interestingly, our data suggested that dsMyoD inhibited proliferation of rat ADSCs during the process of myogenic differentiation. It has been reported that MyoD could trigger or induce other factor to initiate cell cycle exit when it mediates cell differentiation (15).

The ADSCs applied in present study were isolated from adult rat adipose tissue (13). Use of ADSCs as our target cell is based on the following considerations. Firstly, ADSCs are abundant in animal or human body and can be obtained with minimal invasion. Furthermore, ADSCs had the ability of multipotent differentiation, long-term proliferation and self-renewal (28,29). Finally, the process of isolation, identification and culture of the cells was easily performed. In summary, ADSCs acts as an economic, applicable and feasible method for tissue engineering and regenerative medicine.

The main limitation of our study is the lack of evidence in vivo which would otherwise compare the difference between ADSCs and dsMyoD treated ADSCs for the treatment of SUI in a rat SUI model. Besides, it would be more persuasive if similar results could be achieved in other cell lineages, such as mature adipocytes.

## CONCLUSIONS

Our findings showed that dsMyoD could induce transcription factor MyoD expression in rat ADSCs and promoted their differentiation into myoblasts in vitro. Although the definite mechanisms of RNAa remain unclear and numerous targets need to be screened so as to activate a particular promoter, RNAa may still offer a promising approach as therapeutics for SUI by activating MyoD.
